# HTLV-1 uveitis

**DOI:** 10.3389/fmicb.2012.00270

**Published:** 2012-07-24

**Authors:** Koju Kamoi, Manabu Mochizuki

**Affiliations:** Department of Ophthalmology and Visual Science, Graduate School of Medical and Dental Sciences, Tokyo Medical and Dental University, Tokyo, Japan

**Keywords:** HTLV-1, uveitis, ocular inflammation, CD4^+^ T cell, T cell clone

## Abstract

Human T cell lymphotropic virus type 1 (HTLV-1) is the first retrovirus described as a causative agent of human disease. Following adult T cell leukemia/lymphoma and HLTV-1-associated myelopathy/tropical spastic paraparesis, HTLV-1 uveitis (HU) has been established as a distinct clinical entity caused by HTLV-1 based on seroepidemiological, clinical, and virological studies. HU is one of the most common causes of uveitis in endemic areas of Japan and can be a problematic clinical entity all over the world. HU occurs with a sudden onset of floaters and foggy vision, and is classified as an intermediate uveitis. Analysis of infiltrating cells in eyes with HU revealed that the majority of infiltrating cells were CD3^+^ T cells, but not malignant cells or leukemic cells based on their T cell receptor usage. HTLV-1 proviral DNA, HTLV-1 protein, and viral particles were detected from infiltrating cells in eyes with HU. HTLV-1-infected CD4^+^ T cell clones established from infiltrating cells in eyes with HU produced large amounts of various inflammatory cytokines, such as IL-1, IL-6, IL-8, TNF-α, and interferon-γ. Taken together, HU is considered to be caused by inflammatory cytokines produced by HTLV-1-infected CD4^+^ T cells that significantly accumulate in eyes; therefore, topical and/or oral corticosteroid treatment is effective to treat intraocular inflammation in patients with HU. Further investigation is needed to establish a specific treatment for HU.

## INTRODUCTION

Retrovirus was first described in the 1970s (Temin and Baltimore, 1972), but its causal relationship with human diseases was not identified until the early 1980s when human T cell lymphotropic virus type 1 (HTLV-1) was identified as an etiologic agent of adult T cell leukemia/lymphoma (ATL; Poiesz et al., 1980; Hinuma et al., 1981; Yoshida et al., 1984). After the discovery of the link between HTLV-1 and ATL, HLTV-1 was also found to be a causal agent of HTLV-1-associated myelopathy/tropical spastic paraparesis (HAM/TSP; Gessain et al., 1985; Osame et al., 1986) and HTLV-1 uveitis (HU; Mochizuki et al., 1992a,b,c).

HTLV-1 uveitis, the third clinical entity of HTLV-1 infection, was established by a series of studies in the highly endemic area of southern Kyushu, Japan. Clinical case reports from this area suggested possible associations of HTLV-1 carriers with various ocular manifestations (Ohba et al., 1989). In the 1990s, the first set of evidence that indicated the causative implication of HTLV-1 in uveitis was reported by Mochizuki and colleagues. They showed clinical and laboratory data consisting of seroepidemiology, clinical features, detection of proviral DNA and mRNA of HTLV-1 from ocular tissues, and detection of viral particles from T cell clones (TCC) derived from the aqueous humor of the patient (Mochizuki et al., 1992a,b). Since then, it has been well established that uveitis is significantly related to HTLV-1. Here, we review historical findings that contributed to the establishment of the HU entity and recent advancements that deepen our understanding of HU.

## SEROEPIDEMIOLOGY

**FIGURE 1:**
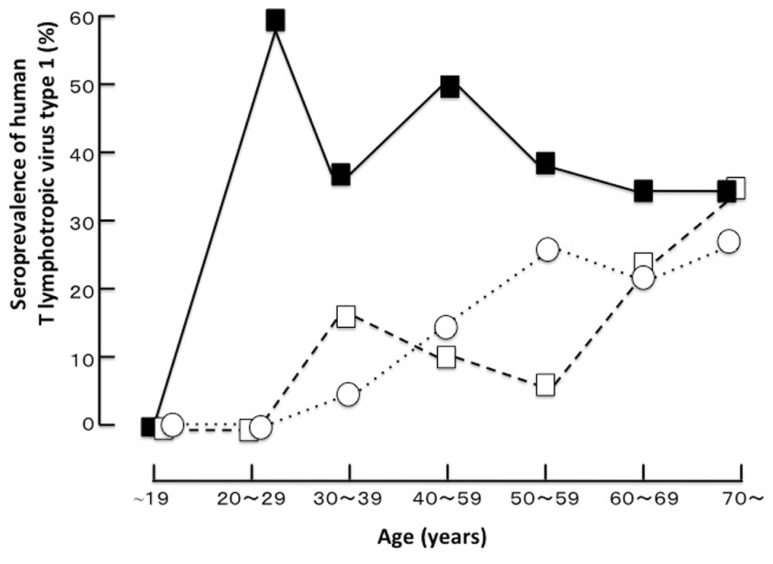
** The age distribution of the seroprevalence of human T cell lymphotropic virus type 1 in a highly endemic area (Miyakonojo, southern Kyushu, Japan; Shirao et al., 1993 with permission)**.■, patients with idiopathic uveitis; □, patients with uveitis from a defined cause; ○, patients with non-uveitic ocular diseases.

HTLV-1 infection is known to have unique geographic distribution and is prevalent in Japan, Melanesia, the Caribbean Islands, Central America, South America, and Central Africa. It is estimated that 20 million people carry the virus worldwide (Watanabe, 2011). This virus is etiologically linked with HU, which is one of the most common causes of uveitis in the endemic area of Japan and can be a problematic clinical entity all over the world (Yoshimura et al., 1993; Takahashi et al., 2000; Merle et al., 2002; Pinheiro et al., 2006; Miyanaga et al., 2009). Uveitis is a sight-threatening inflammatory disorder affecting the intraocular tissues (Forrester, 1991) and is the third leading cause of blindness in developed countries. The etiology of uveitis is categorized as infectious or non-infectious and varies depending on the genetic background of the population and the prevalence of the pathogenic agent in the area. Clinically, the etiology of approximately 30% of cases could not be defined even when careful examinations were performed. A survey comparing the etiologies of uveitis in different areas of Japan demonstrated that the proportion of undefined etiologies was particularly high in southern Kyushu as compared to those in northern Kyushu and Tokyo. Seroepidemiological comparison studies (Mochizuki et al., 1992a,b; Shirao et al., 1993) in these highly endemic and non-endemic areas revealed that the HTLV-1 seroprevalence in patients with idiopathic uveitis was significantly higher than that in the following two control groups: patients with etiology-defined uveitis and patients with non-uveitic ocular diseases (**Figure 1**). This was the first clue suggesting that HTLV-1 infection is significantly related to uveitis. Uveitis is now recognized as a distinct clinical entity related to HTLV-1 and is designated as HU. The seroprevalence of HTLV-1 in the general Japanese population is known to have decreased after serological screening tests of HTLV-1 in blood donors started in 1987, as blood transfusion and breastfeeding from mother to child are major routes of viral transmission (Iwanaga et al., 2009). A recent survey (Miyanaga et al., 2009) in the HTLV-1 endemic region revealed that the most common clinical entity was still HU, followed by Vogt–Koyanagi–Harada disease, sarcoidosis, and others. However, new cases of HU clearly decreased with time, while the prevalence of Vogt–Koyanagi–Harada disease and sarcoidosis has not changed much in the last two decades. The age distribution of HTLV-1 seroprevalence of all patients with uveitis including HU and of patients with uveitis excluding HU showed that the HTLV-1 seroprevalence increased with age in patients of both groups (Yoshimura et al., 1993; Takahashi et al., 2000; Miyanaga et al., 2009). As for the sex, higher prevalence rates were found in women, especially after 40 years of age. HTLV-1 is known to be transmitted by infected lymphocytes in sperm and this may contribute to the higher prevalence of the disease in women than in men (Yoshimura et al., 1993; Takahashi et al., 2000; Miyanaga et al., 2009). As for the prevalence of HU in different parts of the world, the prevalences of HU in Martinique (Merle et al., 2002) and Brazil (Rathsam-Pinheiro et al., 2009) are lower than that in Japan (Yamamoto et al., 1999; Pinheiro et al., 2006). In general, as migration to metropolitan areas is on the rise, the number of HTLV-1 carriers in metropolitan areas (for example, Tokyo) is significantly increasing (Uchimaru et al., 2008), although the number of carriers is still the highest in the endemic areas. In consideration of this evidence, it is estimated that the number of patients with HU is prospectively increasing in metropolitan areas. Therefore, careful examination concerning HU is needed for the diagnosis of uveitis.

## CLINICAL MANIFESTATIONS

A recent report indicated that ocular disturbances may be the first manifestations of HTLV-1 infection to come to clinical attention, in addition to neurologic and rheumatologic signs and symptoms (Poetker et al., 2011). Therefore, all patients presenting for an initial diagnosis should be strictly screened for ocular symptoms. The major symptoms of HU at initial presentation are sudden onset of floaters, foggy vision, and blurred vision. Other symptoms are pain/burning, itching, and foreign body sensation. These symptoms appear in all geographic regions according to studies in Japan, Brazil, and Martinique (Yoshimura et al., 1993; Merle et al., 2002; Pinheiro et al., 2006). Regarding the anatomic diagnosis of uveitis according to the criteria of the International Uveitis Study Group, most patients had an intermediate degree of uveitis with moderate or heavy vitreous opacities (fine cells and lacework-like membranous opacities). The vitreous opacities were the most impressive findings and were accompanied by mild iritis and mild retinal vasculitis, but no uveoretinal lesions (Yoshimura et al., 1993). The ocular inflammation of HU was unilateral or bilateral (Yoshimura et al., 1993; Merle et al., 2002; Pinheiro et al., 2006). An association between HU and Graves’ disease has been reported; HU occurs after the onset of Graves’ disease in all cases (Yamaguchi et al., 1994). The most recent study (Miyanaga et al., 2009) reported a similar incidence of HU after Graves’s disease as that reported by Yamaguchi et al. (1994). Only a few cases of HU develop into HAM/TSP, but no literature has reported that ATL develops in patients with HU during their clinical course. Further patient-tracking research is ongoing to determine whether HU is a risk factor for the development of ATL or HAM/TSP.

## DIAGNOSIS

Considering seroepidemiological and clinical studies, the diagnosis of HU should be based on seropositivity for HTLV-1 with no systemic evidence of HTLV-1-related diseases (such as ATL or HAM/TSP) and exclusion of other uveitis entities with defined causes. Therefore, all clinical entities of uveitis with defined causes should be excluded by careful ophthalmic and systemic examinations. Patients with HU should not have ophthalmic and systemic symptoms that are compatible with other types of uveitis such as Behçet’s disease, Vogt–Koyanagi–Harada syndrome, and sarcoidosis.

## PATHOGENESIS

**FIGURE 2:**
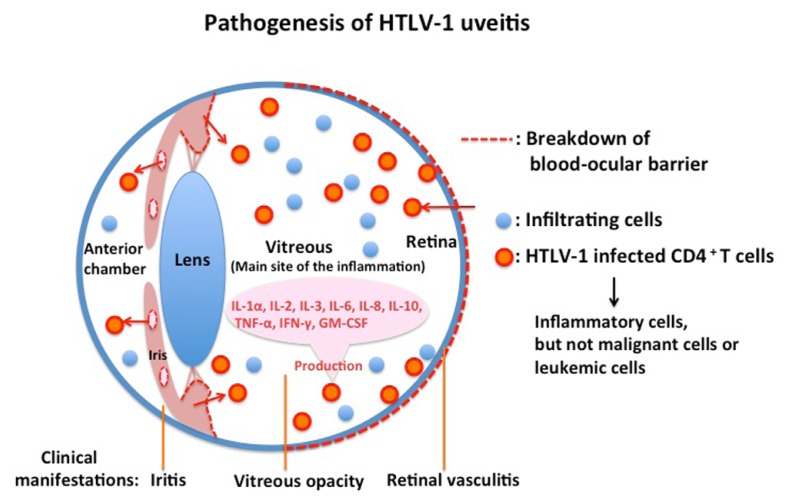
**The majority of infiltrating cells in eyes with HTLV-1 uveitis are inflammatory cells and not malignant or leukemic cells.**HTLV-1 uveitis is caused by inflammatory cytokines produced by HTLV-1-infected CD4^+^ T cells that significantly accumulate in the eyes of patients.

Eye research has progressed significantly in accordance with the development of modern molecular biological technology, such as the polymerase chain reaction and flow cytometry. Many fundamental findings have been obtained in the study of HU pathogenesis. The cells floating in the anterior chamber of the eye with HU consisted of lymphocytes with a small proportion of macrophages. No malignant cells or leukemic cells were detected in the aqueous humor of the patients with HU (Masuoka et al., 1995). The majority of infiltrating cells in the aqueous humor of patients with HU were CD3^+^ T cells (Ono et al., 1997). Analysis by polymerase chain reaction of ocular-infiltrating cells revealed that HTLV-1 proviral DNA was detected in almost all patients with HU. However, proviral DNA was not detected in patients with uveitis of other defined etiology who were seropositive for HTLV-1. These data suggest that HTLV-1-infected cells are present at the local site of HU (Ono et al., 1997). Furthermore, expression of viral mRNA was detected by reverse transcriptase-polymerase chain reaction from the inflammatory cells in the aqueous humor. More direct evidence of HTLV-1 in the pathogenesis of HU has been provided by using TCC derived from intraocular tissues of eyes with HU. Proviral DNA of HTLV-1 was identified in TCC from the ocular fluid (Sagawa et al., 1995). Immunohistochemical staining showed that HTLV-1 env and gag proteins were detectable in HTLV-1 provirus-positive TCC. Furthermore, electron microscopic observation of the TCC identified HTLV-1 virus particles, the mean diameter of which was 102 nm (Sagawa et al., 1995). Most HTLV-1-infected TCC had a CD3^+^CD4^+^CD8^-^ phenotype and had polyclonal TCRα usage (Sagawa et al., 1995). The HTLV-1-infected TCC produced significant amounts of IL-1α, IL-2, IL-3, IL-6, IL-8, IL-10, TNF-α, IFN-γ, and GM-CSF, which are potent cytokines capable of inducing immune reactions and inflammation at the intraocular tissue level (Sagawa et al., 1995). These data suggest that cytokine production by HTLV-1-infected T cells in intraocular tissues is responsible for intraocular inflammation, i.e., uveitis (**Figure 2**). In addition to this molecular biological/immunological evidence, virological research supported the pathogenicity of HTLV-1 in the eye by the following three pieces of evidence: (1) the HTLV-1 provirus load in patients with HU is significantly higher than that in asymptomatic carriers without uveitis (Ono et al., 1995); (2) the proviral load in peripheral blood mononuclear cells correlates with the intensity of intraocular inflammation (Ono et al., 1998); and (3) the proviral load in the eyes of patients with HU is significantly higher than that present in peripheral blood mononuclear cells (Ono et al., 1997). Serologic data showed that the antibody level against HTLV-1 in patients with HU was similar to that in asymptomatic carriers of HTLV-1, but was lower than that in patients with HTLV-1-associated myelopathy (Mochizuki et al., 1992b). Antibody to the virus in the aqueous humor was also detected in all tested samples from patients with HU. Flow cytometry analysis indicated that the CD4 fraction was elevated and the CD8 fraction was decreased in peripheral lymphocytes from patients with HU, thereby elevating the CD4/8 ratio in the HU group as compared with the seronegative group. Furthermore, the CD25 fraction of T lymphocytes with expression of interleukin 2 receptors was significantly elevated in patients with HU. The serum levels of soluble interleukin 2 receptors (sIL2R or sCD25) were also significantly higher in patients with HU than in seronegative healthy controls (Yoshimura et al., 1993). Taken together, these laboratory data suggest that the immune-mediated mechanism, particularly involving CD4^+^ T cells, plays a critical role in the pathogenesis of HU.

## THERAPY

Immunopathogenesis studies of HU showed that the majority of ocular-infiltrating cells are inflammatory cells, but not malignant cells. Also, a series of studies showed that HU is caused by inflammatory cytokines produced by HTLV-1-infected CD4^+^ T cells that significantly accumulate in the eyes of the patients. Furthermore, the addition of corticosteroids in the culture medium suppressed the cytokine production (Sagawa et al., 1995). Therefore, corticosteroid treatment is effective for treating the intraocular inflammation of patients with HU by suppressing the cytokine production of HTLV-1-infected CD4^+^ T cells. Clinical management should be performed according to the degree of ocular inflammation. HU with a mild degree of ocular inflammation can be managed by topical non-corticosteroidal or corticosteroidal anti-inflammatory drugs. A sub-Tenon’s injection of corticosteroids may be used when the patients have moderate inflammatory activity in the vitreous cavity. If the vitreous inflammatory activity and the retinal vasculitis are severe, oral corticosteroids should be given, but the long-term administration of a systemic corticosteroid should be avoided. In most cases, intraocular inflammation is markedly improved with these therapies and complete remission is achieved. The visual prognosis for cases of HU is generally good with these corticosteroid treatments, although approximately 60% of patients experience recurrences of uveitis (Yoshimura et al., 1993).

## CONCLUSION

We reviewed the seroepidemiological, clinical, molecular biological, and virological studies that established the HU entity and clarified the immunopathogenesis and the clinical management of HU. Corticosteroid is the only effective treatment for HU to suppress the cytokines produced by infiltrating HTLV-1-infected cells; however, it is unknown whether long-term corticosteroid treatment adversely affects patients with HU. Many mechanisms in HU remain unclear, such as how HTLV-1-infected CD4^+^ cells break down the ocular blood barrier and why the vitreous humor is the major site of inflammation (**Figure 2**). We may be able to find more effective treatments if we can understand the mechanism of HU in more detail. Recent studies have shown new insights into HTLV-1 infection and pathogenesis by pursuing the molecular functions of HTLV-1 basic leucine zipper factor and Tax (Yasunaga and Matsuoka, 2011). However, few studies have been conducted to apply these new findings to HU research. Further investigation is needed to establish a specific treatment for HU. HU results from HTLV-1 infection; therefore, the most important means of preventing this disease is by spreading the knowledge about HTLV-1.

## Conflict of Interest Statement

The authors declare that the research was conducted in the absence of any commercial or financial relationships that could be construed as a potential conflict of interest.
